# A Study of Tongue and Pulse Diagnosis in Traditional Korean Medicine for Stroke Patients Based on Quantification Theory Type II

**DOI:** 10.1155/2013/508918

**Published:** 2013-04-11

**Authors:** Mi Mi Ko, Tae-Yong Park, Ju Ah Lee, Byoung-Kab Kang, Jungsup Lee, Myeong Soo Lee

**Affiliations:** ^1^Medical Research Division, Korea Institute of Oriental Medicine, 1672 Yuseongdae-ro, Yuseong-gu, Daejeon 305-811, Republic of Korea; ^2^Department of Oriental Rehabilitation Medicine, Korean National Rehabilitation Center, 58 Samgaksan-ro, Gangbuk-gu, Seoul 142-884, Republic of Korea

## Abstract

In traditional Korean medicine (TKM), pattern identification (PI) diagnosis is important for treating diseases. The aim of this study was to comprehensively investigate the relationship between the PI type and tongue diagnosis or pulse diagnosis variables. The study included 1,879 stroke patients who were admitted to 12 oriental medical university hospitals from June 2006 through March 2009. The status of the pulse and tongue was examined in each patient. Additionally, to investigate relatively important indicators related to specialist PI, the quantification theory type II analysis was performed regarding the PI type. In the first axis quantification of the external criteria, the Qi-deficiency and the Yin-deficiency patterns were located in the negative direction, while the dampness-phlegm (DP) and fire-heat patterns were located in the positive direction. The explanatory variable with the greatest impact on the assessment was a fine pulse. In the second axis quantification, the external criteria were divided into either the DP or non-DP patterns. The slippery pulse exhibited the greatest effect on the division. This study attempted to build a model using a statistical method to objectively quantify PI and various indicators that constitute the unique diagnosis system of TKM. These results should assist the development of future diagnostic standards in stroke PI.

## 1. Introduction

 Traditional Korean medicine (TKM) uses a unique diagnostic system of pattern identification (PI) based on the indicated reactions of the body to disease [1]. TKM uses the four methods of diagnosis, which include diagnosis by observation, diagnosis by hearing and smelling, diagnosis by interrogation, and diagnosis by palpation [1, 2]. One of the typical methods of diagnosis by observation is tongue diagnosis, which is the evaluation of a disease by observing the tongue. This method is actively used to examine the causes, properties, and affected areas of a disease and to determine the prognosis of a disease by observing changes in a patient's tongue characteristics and tongue coating. The most representative method of diagnosis involves palpation and is known as pulse diagnosis. Pulse diagnosis is an examination technique in which the doctor directly palpates the pulses on both wrists of a patient to evaluate the properties and condition of the pulses. A patient's condition and disease are diagnosed according to the palpation of the pulse, a treatment plan is chosen, and the effectiveness of the selected treatment is determined by comparing the pulses before and after the treatment.

The definition of “pattern” in TKM refers to the overview of each step in the course of a disease and consists of a combination of correlations between several symptoms and signs [3]. Previous reports have described the PI process for differentiating stroke victims with four TKM types: the fire-heat pattern, dampness-phlegm pattern, Yin-deficiency pattern, and Qi-deficiency pattern [4–6]. However, the meaning of a pattern is often ambiguous in clinical practice, and it is difficult to objectively arrange or accumulate the clinical data regarding these patterns or perform a systematic analysis of the results. TKM emphasizes PI as an expedient for treatment by specifying the nature of a disease, but collecting quantitative data for the assessments is difficult because the assessments rely on the patient's subjective descriptions and the doctor's subjective judgments.

The quantification theory applied in this study follows the theory of the quantification of qualitative data developed by Hayashi in Japan after 1950 [7]. This quantification theory consists of four types classified as I, II, III, and IV. The quantification of the type II analysis used in this study uses a canonical correlation analysis or a canonical discriminant analysis when both the external criteria and explanatory variables are qualitative [7, 8]. This quantification method examines the correlation among the categories of dependent variables and the categories of covariates, and it is very useful in the medical research field for the estimation, diagnosis, prognosis, and evaluation of epidemiological factors [9]. Previous studies have used this quantification method in TKM [10–12]. Recently, we conducted a quantification theory method to investigate the relationship between PI types and its properties for using stroke patients from TKM hospitals [12].

Hayashi's quantification type II analysis was performed in the current study using variable data from tongue diagnosis and pulse diagnosis. The data were collected from case report forms (CRFs) with a focus on PI for stroke patients at 12 TKM hospitals throughout the nation [5]. Using this analysis, we aimed to comprehensively investigate the correlations among the PI diagnosed by a specialist, tongue diagnosis, and pulse diagnosis.

## 2. Methods

### 2.1. Study Subjects

**Figure 1:**
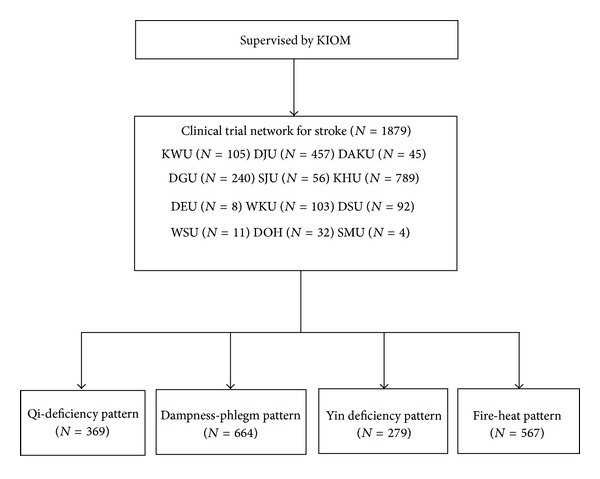
Flow chart showing patient enrollment in study. KIOM, Korean Institute of Oriental Medicine; KWU, Kyung Won Oriental Medical Hospital; DJU, Dae Jeon Oriental Medical Hospital; DAKU, Dae Gu Hanny University Medical Center; DGU, Dong Guk International Hospital; SJU, Sang Ji Oriental Medical Hospital; KHU, Kyung Hee Oriental Medical Hospital; DEU, Dong Eui Oriental Medical Hospital; WKU, Won Kwang Oriental Medical Hospital; DSU, Dong Sin Oriental Medical Hospital; WSU, Woo Suk Oriental Medical Hospital; DOH, Dong Seo Oriental Medical Hospital; SMU, Se Myeong Oriental Medical Hospital; QD, Qi-deficiency pattern; DP, dampness-phlegm pattern; YD, Yin-deficiency pattern; FH, fire-heat pattern.

As part of the research on the standardization of oriental medicine stroke PI, the Korea Institute of Oriental Medicine (KIOM) collected stroke data for stroke PI from June 2006 to March 2009 [4–6]. The data were obtained from 12 TKM hospitals and oriental medical universities throughout the nation (Figure 1). The eligibility inclusion criteria included enrolled stroke patients within 30 days of the onset of symptoms as confirmed by imaging diagnosis, such as computerized tomography (CT) or magnetic resonance imaging (MRI). The exclusion criteria included traumatic stroke patients, such as patients with subarachnoid, subdural, or epidural hemorrhages. Two clinical specialists who were well trained in the standard operation procedures (SOPs) simultaneously examined the patterns of identification for a patient, and the symptoms present within 24 hours of the time of examination were used as the basis for analysis. The specialists had at least 3 years of clinical experience with stroke patients after finishing regular college education for TKM for 6 years. Based on the symptoms described by the patients, the doctor's objective judgments were recorded. Among the data collected in this manner, 1,879 data entries with PI by the 2 specialists were used for the analysis. All the patients provided written informed consent under procedures approved by the institutional review boards (IRB).

### 2.2. Data Processing and Analysis

The chi-square test and Fisher's exact test were used for the analysis of discrete variables. The Kolmogorov-Smirnov method was used to test the normality of the continuous variables, which were analyzed using one-way ANOVA (as a parametric method) and the Kruskal-Wallis test (as a nonparametric method). The examination parameters were extracted from CRFs for the standardization of stroke diagnosis developed by an expert committee organized by the KIOM [4–6]. Each patient received an examination of the status of the tongue and pulse, tongue color (pale, pale-red, red, or bluish purple), fur color (white fur or yellow fur), fur quality (thick fur or dry fur), special tongue appearance (teeth marked, enlarged, mirror, or spotted), pulse location (floating or sunken), pulse rate (slow or rapid), pulse force (strong or weak), and pulse shape (fine, slippery, rough, or surging). The analysis was performed by converting the index measurements obtained using the 3-point scale in which 3 = very much, 2 = much, and 1 = not much into a 2-point scale in which 1 = yes and 0 = no. The information of the measurement variables is shown in Supplementary Table 1 (see Supplementary Material available online at http://dx.doi.org/10.1155/2013/508918). The PI data assessed in this study included the results of the 4 PIs, which were measured as fire-heat (FH), dampness-phlegm (DP), Qi-deficiency (QD), and Yin-deficiency (YD) patterns. These assessments were given individually without discussions among the specialists. A total of 1,879 stroke patients received a PI assessment with the same opinions by specialists with the following distribution: FH pattern (*n* = 567), DP pattern (*n* = 664), YD pattern (*n* = 279), and QD pattern (*n* = 369) (Figure 1).

The quantification type II theory, which is a multivariate analysis, is suitable when predicting qualitative values as the external criteria variable based on the information concerning the qualitative explanatory variables of each subject [7, 8]. Most of the variables in this study are categorical, and the quantification type II theory based on canonical correlation analysis (CCA) was applied to identify the relationship between two sets of multidimensional variables including the 4 types of PI and the tongue/pulse diagnosis variables. Concurrence between the 2 specialists served as the external criteria, and the tongue diagnosis and pulse diagnosis variables served as the explanatory variables (see Supplementary Table 2).

The CCA was conducted first for the quantification type II theory. Raw canonical coefficients that were used for the quantification type II theory were derived from the CCA. After finding the raw canonical coefficients, the quantification value was calculated. We calculated the centering value (*c*_*p*_) (1) based on the raw canonical coefficients and obtained the categorical score (*s*_*pq*_) using a simple formula (2) as

(1) $$\begin{array}{c} c_{p}=\frac{\sum_{q=1}^{n_{p}}a_{pq}\times x_{pq}}{\sum_{q=1}^{n_{p}}x_{pq}}, \end{array}$$

where *x*_*pq*_ is the frequency of level *q* of the *p*th category variable, *a*_*pq*_ is the associated with level *q* of each *p*th category variable, and *n*_*p*_ is the total number of *p*th category variables.

Consider the following:

(2) $$\begin{array}{c} s_{pq}=a_{pq}-c_{p}, \end{array}$$

where *s*_*pq*_ is the categorical score (quantification value) of level *q* of the *p*th category variable.

The quantification range represents the difference between the maximum quantification value and the minimum quantification value, and it is used as an indicator with the partial correlation coefficient to show the importance of each explained variance.

SAS (Version 9.1.3, SAS Institute Inc., Cary, NC, USA) was used for the statistical analysis.

## 3. Results

The general characteristics of the study subjects are shown in Table 1.

Throughout the CCA for the quantification type II analysis, the canonical correlation coefficient for *Y*1 and *X*1 in the first axis was 0.585, and the canonical correlation coefficient of *Y*2 and *X*2 in the second axis was 0.555 (Table 2). The third axis was not considered because the canonical correlation coefficient in the third axis was relatively small compared with the other axes. The first two axes explain 91.54% of the total data (first axis: 49.29%, second axis: 42.25%).

The first axis quantification for the external criteria consisted of assessments of (−) QD, YD, DP, and FH (+) axes. The QD and the YD were located at (−) quantification values of −1.5990 and −0.6004, respectively, while the DP and the FH values were located at (+) 0.1103 and 1.2069, respectively (Table 3). Based on the quantification range, the explained variance with the highest correlation corresponds with the fine pulse state. The assessments of fine pulse (−0.3731) were related to the QD and the YD, whereas the assessments without fine pulse (0.0958) were related to the DP and the FH patterns. Pale tongue (a type of tongue diagnosis) also demonstrated considerable significance as an explanatory factor; the demonstrations of pale tongue (−0.1223) was predicted the diagnoses of QD and YD. Furthermore, yellow fur (a type of tongue diagnosis) followed by weak pulse (a type of pulse states) and surging pulse (a type of pulse state) was shown to be significant explanatory factors (Tables 4 and 5).

Regarding the external criteria, the second axis quantification included the contrast between the (−) DP and non-DP (+) assessments (Table 3). Based on the quantification range, the factor with the highest correlation was the slippery pulse. The assessments of slippery pulse (0.0582) were related to the DP quality. Spotted tongue (a type of tongue diagnosis) also demonstrated considerable significance as an explanatory factor; cases with spotted tongue (0.1710) were correlated with the DP readout. Pale tongue (a type of tongue diagnosis) and dry fur were significant explanatory factors (Tables 4 and 5).

**Figure 2:**
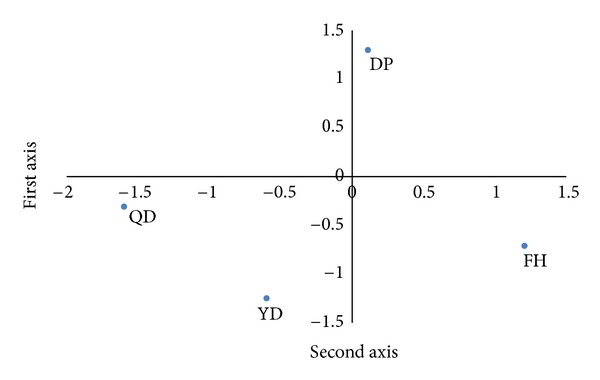
Quantification plot of external criterion. FH, fire-heat; DP, dampness-phlegm; QD, Qi-deficiency; YD, Yin-deficiency patterns.

**Figure 3:**
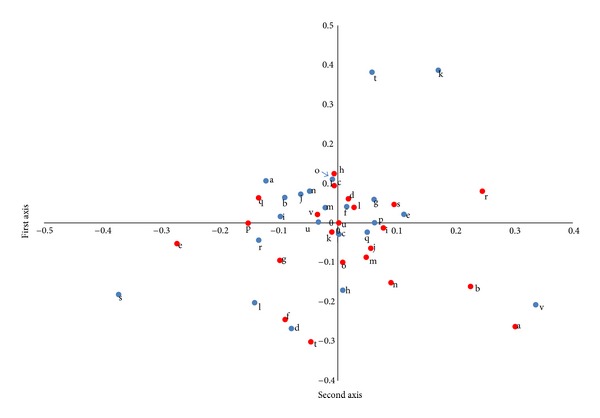
Quantification plot of independent variables. a, pale; b, pale red; c, red; d, bluish purple; e, yellow fur; f, white fur; g, thick fur; h, dry fur; i, teeth marked; j, enlarged; k, spotted; l, mirror; m, floating; n, sunken; o, slow; p, rapid; q, strong; r, weak; s, fine; t, slippery u, rough; v, surging. Blue color and red color denote yes, and no respectively.

Figure 2 shows the quantification plot using the quantification values of the first axis and the second axis for the 4 types of PI as the external properties, and Figure 3 shows the quantification plot using the quantification values of the first axis and the second axis for the indicators as explanatory variables.

## 4. Discussion

For the diagnosis and treatment of stroke, TKM has established a principle of diagnosis based on various patient symptoms using a method known as PI to identify symptoms resulting from a diseased condition or the development of a disease in the human body [1]. The process of PI integrates many symptoms, and tongue diagnosis and pulse diagnosis serve as important disease indicators [2].

As a part of the SOPI-STROKE project, the KIOM recently created the Korean standard PI for stroke (K-SPI) for use in Korea [4–6]. This system consists of 4 types of PI (FH, DP, YD, and QD) and 44 types of indicators [5]. The FH pattern is characterized by any symptom of heat or fire that is contracted externally or engendered internally. The DP pattern is characterized by impeding Qi movement and its turbidity, heaviness, stickiness, and downward-flowing properties. The QD pattern is characterized by Qi deficiency with diminished internal organ function, which is marked by shortness of breath, lassitude, listlessness, spontaneous sweating, a pale tongue, and a weak pulse. The YD pattern is characterized by Yin deficiency with diminished moistening and the inability to restrain yang, which is usually manifested as fever [4–6]. 

This study aimed to examine the correlation between stroke PI and tongue diagnosis or pulse diagnosis variables using a quantitative analysis. The quantification theory type II is suitable when predicting the qualitative data as the external criteria variable based on the information concerning the qualitative explanatory variables of each subject. Shin [10] suggested the quantification theory type II method to quantify the knowledge obtained in the process of TKM diagnoses using stroke data but used a small sample size of only 45 stroke patients. No pulse information and few tongue indicators were obtained, which makes it difficult to generalize the results. In 2010, we conducted the quantification theory type II method to investigate the relationship between PI types and properties using 835 stroke patients from TKM hospitals [12]. The pulse status and tongue status were evaluated along with other items (e.g., headaches, dizziness, facial complexion items, skin items, urine items, etc.). However, the discriminative estimation of the YD pattern is relatively low compared with other patterns. In this study, as a result of the first axis quantification of the external criteria, the QD and the YD were located in the negative direction, while the DP and FH patterns were located in the positive direction, and the locations were used to differentiate between excess and deficiency. There are many methods for PI diagnosis in TKM, and PI diagnosis according to the eight guiding principles is the basis for all the other methods. It involves the categorization of a patient's condition according to four opposing pairs of principles: interior/exterior, heat/cold, excess/deficiency, and the overall summary principles of Yin-Yang. Excess and deficiency indicate the relative strength of the pathogenic factor and the Qi [13, 14].

 The explanatory variable with the greatest impact on the assessment was a pulse diagnosis variable known as the fine pulse, and the diagnosis was related to both types of deficiencies. The tongue diagnosis variable of pale tongue was also highly relevant as an explanatory factor. Fine pulse and pale tongue are important factors for the diagnosis of the QD pattern according to K-SPI-III [5].

As a result of the second axis quantification, the external criteria were divided into either the DP or non-DP patterns. The DP and non-DP cases can be distinguished by the presence or absence of a pathological product known as DP. The DP patterns, which is classified by etiology, is a combination of phlegm and internal dampness causing disease. Slippery pulse was the explanatory variable with the greatest effect on the division. Kim's study [2] also reported that slippery pulse was the most potent factor for determining the DP pattern.

Comparing the results with the K-SPI-III confirms that the results agree in many aspects with the exception of only few elements. The result shows that specific tongue and pulse indicators are important factors for the difficult diagnosis of excess/deficiency.

 Actual diagnoses are performed using the pulse diagnosis and tongue diagnosis methods and by pooling information from the four diagnostic methods. Therefore, future studies should perform these types of analyses by considering pooled information from the four diagnostic methods. Tongue and pulse diagnoses depend on the clinician's experience and knowledge and a variety of environmental factors. It is essential to establish an objective diagnostic standard for tongue and pulse diagnoses such as detailed SOPs or other diagnosis tools.

 Although there were some limitations, the results of this study support the objectivity of the theories of TKM. In addition, this study can be considered significant because it attempted to build a model using a statistical method to objectively quantify PI and various indicators that constitute the unique diagnosis system of TKM. Furthermore, the results of this study should be very helpful in the selection, development, and modification of future diagnostic standards in TKM for stroke PI.

## Supplementary Material

Supplementary table 1 (This summary is the information of the measurement variables; frequency of tongue and pulse diagnosis variables)
Supplementary table 2 (This information is description of variables used in this study.)

## Figures and Tables

**Table 1:** Demographic parameters of study subjects.

Characteristics	Fire-heat	Dampness- phlegm	Qi deficiency	Yin deficiency	*P *
*N *	567	664	369	279	
Sex (M/F)	425/142	306/358	121/248	128/151	∗∗
Age (Mean ± SD)	65.50 ± 11.82	66.41 ± 11.13	67.31 ± 11.89	69.54 ± 11.84	∗∗
Weight (kg) (Mean ± SD)	65.09 ± 10.55	63.77 ± 10.59	56.63 ± 9.21	57.42 ± 11.10	∗∗
Height (cm) (Mean ± SD)	164.07 ± 8.42	160.37 ± 8.71	157.51 ± 8.11	158.54 ± 12.14	∗∗
BMI (Mean ± SD)	24.23 ± 2.93	24.74 ± 3.20	22.78 ± 2.97	22.79 ± 4.09	∗∗
WHR (Mean ± SD)	0.95 ± 0.09	0.94 ± 0.08	0.92 ± 0.10	0.93 ± 0.13	∗∗
WC (cm) (Mean ± SD)	88.62 ± 9.36	88.77 ± 9.57	84.46 ± 8.97	83.00 ± 9.30	∗∗
HC (cm) (Mean ± SD)	94.10 ± 9.21	94.27 ± 9.28	91.67 ± 9.28	90.21 ± 10.62	∗∗
TOAST classification					
LAA	153	138	62	66	∗
CE	40	31	23	19	
SVO	271	402	215	138	
SOE	5	11	7	8	
SUE	23	29	14	10	
Hypertension (yes, no)	332/233	419/243	217/147	161/118	NS
Hyperlipidemia (yes, no)	77/478	86/572	44/320	24/249	NS
DM (yes, no)	144/417	192/469	97/268	66/211	NS
Smoking (none/stop/active)	220/127/220	387/88/186	239/44/85	154/42/83	∗∗
Drinking (none/stop/active)	230/74/263	389/60/212	223/36/109	151/29/99	∗∗

BMI: body mass index. WHR: waist hip ratio. WC: waist circumference. HC: hip circumference. TOAST: trial of ORG 10172 in acute stroke treatment. LAA: large-artery atherosclerosis. CE: cardioembolism. SVO: small-vessel occlusion. SOE: stroke of other etiology. SUE: stroke of undetermined etiology. DM: diabetes mellitus. NS: not significant. **P < .0001.*P < .01.

**Table 2:** Results of the canonical correlation analysis.

Variate number	Canonical correlation	Approximate standard error	Eigenvalue	Proportion	Cumulative	*P *
1	0.5848	0.0152	0.5193	0.4929	0.4929	∗∗
2	0.5550	0.0159	0.4452	0.4225	0.9154	∗∗
3	0.2861	0.0212	0.0891	0.0846	1	∗∗

**P < .0001.

**Table 3:** Calculated result by Hayashi's quantification method type 2: external criterion.

External criterion	First axis			Second axis	
	Category scores	Range		Category scores	Range
Fire-heat	1.2069	2.8059		−0.7094	2.5485
Dampness-phlegm	0.1103			1.3007	
Qi deficiency	−1.5990			−0.3071	
Yin deficiency	−0.6004			−1.2478	

**Table 4:** Calculated result by Hayashi's quantification method type 2: tongue indicators.

Variable	First axis			Second axis		
	Category scores	Range	Partial correlation^‡^	Category scores	Range	Partial correlation^‡^
Pale						
y	−0.1223	0.4241	0.0871	0.1065	0.3693	0.0741
n	0.3018			−0.2628		
Palered						
y	−0.0903	0.3161	0.0657	0.0644	0.2254	0.0457
n	0.2258			−0.1610		
Red						
y	0.0019	0.0080	0.0017	−0.0288	0.1230	0.0250
n	−0.0061			0.0942		
Bluish purple						
y	−0.0790	0.0970	0.0183	−0.2676	0.3285	0.0605
n	0.0180			0.0609		
Yellow fur						
y	0.1125	0.3863	0.1504	0.0216	0.0741	0.0285
n	−0.2738			−0.0525		
White fur						
y	0.0151	0.1048	0.0460	0.0411	0.2858	0.1215
n	−0.0897			−0.2447		
Thick fur						
y	0.0617	0.1604	0.0854	0.0593	0.1542	0.0802
n	−0.0987			−0.0949		
Dry fur						
y	0.0085	0.0147	0.0071	−0.1706	0.2952	0.1381
n	−0.0062			0.1245		
Teeth marked						
y	−0.0978	0.1753	0.0745	0.0161	0.0289	0.0120
n	0.0775			−0.0128		
Enlarged						
y	−0.0632	0.1192	0.0519	0.0726	0.1371	0.0582
n	0.0561			−0.0645		
Spotted						
y	0.1710	0.1812	0.0234	0.3865	0.4097	0.0516
n	−0.0102			−0.0232		
Mirror						
y	−0.1415	0.1691	0.0376	−0.2023	0.2416	0.0523
n	0.0275			0.0393		

^‡^Partial correlation coefficient. y and n denote yes and no, respectively.

**Table 5:** Calculated result by Hayashi's quantification method type 2: pulse indicators.

Variable	First axis			Second axis		
	Category scores	Range	Partial correlation^‡^	Category scores	Range	Partial correlation^‡^
Floating						
y	−0.0214	0.0697	0.0316	0.0387	0.1259	0.0557
n	0.0483			−0.0872		
Sunken						
y	−0.0480	0.1384	0.0621	0.0804	0.2319	0.1012
n	0.0904			−0.1515		
Slow						
y	−0.0092	0.0175	0.0071	0.1103	0.2102	0.0833
n	0.0083			−0.0998		
Rapid						
y	0.0625	0.2152	0.1085	0.0003	0.0011	0.0005
n	−0.1526			−0.0008		
Strong						
y	0.0501	0.1850	0.0842	−0.0236	0.0870	0.0387
n	−0.1349			0.0634		
Weak						
y	−0.1347	0.3806	0.1552	−0.0439	0.1240	0.0499
n	0.2459			0.0801		
Fine						
y	−0.3731	0.4688	0.1954	−0.1815	0.2281	0.0941
n	0.0958			0.0466		
Slippery						
y	0.0582	0.1042	0.0573	0.3816	0.6828	0.3447
n	−0.0460			−0.3012		
Rough						
y	−0.0331	0.0347	0.0088	0.0020	0.0021	0.0005
n	0.0016			−0.0001		
Surging						
y	0.3368	0.3715	0.1253	−0.2073	0.2286	0.0756
n	−0.0347			0.0213		

^‡^Partial correlation coefficient. y and n denote yes and no, respectively.

## References

[1] Deng T (1984). *Basic Theory of Traditional Chinese Medicine*.

[2] Kim HJ, Bae HS, Park SU, Moon SK, Park JM, Jung WS (2011). Clinical approach to the standardization of oriental medical diagnostic pattern identification in stroke patients. *Evidence-Based Complementary and Alternative Medicine*.

[3] Choi SM, Yang KS, Choi SH (1997). Standardization and unification of the terms and conditions used for diagnosis in oriental medicine (III). *Korea Journal of Oriental Medicine*.

[4] Park TY, Lee JA, Cha MH (2012). The fundamental study of the standardization and objectification of pattern identification in traditional Korean medicine for stroke (SOPI-Stroke): an overview of phase 1. *European Journal of the Integrative Medicine*.

[5] Lee JA, Lee JS, Kang BK (2011). Report on the Korean standard pattern identification for the stroke-III. *Korean Journal of Oriental Internal Medicine*.

[6] Lee JA, Park TY, Lee JS (2012). Developing indicators of pattern identification in patients with stroke using traditional Korean medicine. *BMC Research Notes*.

[7] Hayashi C (1950). On the quantification of qualitative data from the mathematico-statistical point of view—an approach for applying this method to the parole prediction. *Annals of the Institute of Statistical Mathematics*.

[8] Tanaka Y (1979). Review of the methods of quantification. *Environmental Health Perspectives*.

[9] Suzuki T, Kudo A (1979). Recent application of quantification II in Japanese medical research. *Environmental Health Perspectives*.

[10] Shin YK (1997). A study on the quantification for Oriental Medicine Data. *Journal of Statistical Theory & Methods*.

[11] Jeon RH, Lee IS, Kim KK, Kang CW (1999). Study on the quantification method of symptoms and signs for the oriental gynaecology experiments. *Journal of the Korean Data Analysis Socity*.

[12] Ko MM, Lee JS, Kang BK, Oh DS, Bang OS (2010). A study on the pattern identification diagnosis of the stroke using quantification method type II. *Journal of the Korean Data Analysis Socity*.

[13] Yong JK (1996). A view on the standardization process of differentiation system and searched. *Journal of Hyungok Academic Society*.

[14] Maciocia G, Ying ZZ (1994). *The Practice of Chinese Medicine: The Treatment of Diseases with Acupuncture and Chinese Herbs*.

