# Supplementary material for: A Study of Tongue and Pulse Diagnosis in Traditional Korean Medicine for Stroke Patients Based on Quantification Theory Type II
Source: Evid Based Complement Alternat Med. 2013 Apr 11;2013:508918. doi: 10.1155/2013/508918 (PMC3638600; doi:10.1155/2013/508918)
Supplement: Supplementary file 1 — Supplementary table 1 (This summary is the information of the measurement variables; frequency of tongue and pulse diagnosis variables) Supplementary table 2 (This information is description of variables used in this study.) [file 508918.f1.docx]

Supplemental Table 1. Frequency of measurement variables

| Measurement variables | Symbols | Frequency of measurement variable value | | |
| --- | --- | --- | --- | --- |
|  |  | 3 | 2 | 1 |
| Tongue indicators | | | | |
| pale | a | 39 | 457 | 201 |
| pale-red | b | 19 | 684 | 281 |
| red | c | 60 | 554 | 188 |
| bluish purple | d | 4 | 67 | 312 |
| yellow fur | e | 75 | 509 | 240 |
| white fur | f | 75 | 890 | 162 |
| thick fur | g | 149 | 539 | 430 |
| dry fur | h | 78 | 311 | 533 |
| teeth marked | i | 21 | 261 | 356 |
| enlarged | j | 10 | 290 | 338 |
| spotted | k | 2 | 20 | 367 |
| mirror | l | 14 | 56 | 360 |
| Pulse indicators | | | | |
| floating | m | 70 | 540 | 271 |
| sunken | n | 41 | 547 | 312 |
| slow | o | 26 | 306 | 367 |
| rapid | p | 66 | 583 | 266 |
| strong | q | 72 | 698 | 286 |
| weak | r | 55 | 511 | 310 |
| fine | s | 35 | 344 | 1476 |
| slippery | t | 62 | 759 | 1040 |
| rough | u | 7 | 80 | 1767 |
| surging | v | 15 | 158 | 1680 |

3=very much so; 2=much so; 1=not so much;

Supplemental Table 2. Variable description.

| Variable | Description^a^ |
| --- | --- |
| Tongue indicators |  |
| pale | A tongue less red than normal, indicating Qi and blood deficiency |
| pale-red | A tongue of normal color |
| red | A tongue redder than normal, indicating the presence of heat |
| bluish purple | A cyanotic tongue, indicating blood stasis of heat |
| yellow fur | A tongue coating yellow in color |
| white fur | A tongue coating white in color |
| thick fur | A tongue coating where the underlying tongue surface is not visible |
| dry fur | A tongue coating that looks dry and feels dry to the touch |
| teeth marked | A tongue with dental indentations on its margin |
| enlarged | A tongue that is larger than normal, pale in color and delicate |
| spotted | A tongue with red, white or black spots |
| mirror | A completely smooth tongue free of coating, like a mirror |
| Pulse indicators |  |
| floating | A superficially located pulse which can be felt by light touch and grows faint on hard pressure |
| sunken | A deeply located pulse which can only be felt when pressing hard, also called deep pulse |
| slow | A pulse with less than four beats to one cycle of the physician’s respiration, the same as bradycardia |
| rapid | A pulse with more than five or six beats to one cycle of the physician’s respiration, the same as tachycardia |
| strong | A general term for strongly beating pulse |
| weak | A pulse that is deep, soft, thin and forceless |
| fine | A pulse as thin as a silk thread, straight and soft, feeble yet always perceptible upon hard pressure, also called thin/thready pulse |
| slippery | A pulse coming and going smoothly like beads rolling on a plate |
| rough | A pulse coming and going unsmoothly with small, fine, slow joggling tempo like scraping bamboo with a knife |
| surging | A pulse beating like dashing waves with forceful rising and gradual decline, also called flooding pulse |
